# Gravitational waves: search results, data analysis and parameter estimation: Amaldi 10 Parallel session C2

**DOI:** 10.1007/s10714-014-1796-x

**Published:** 2015-01-22

**Authors:** Pia Astone, Alan Weinstein, Michalis Agathos, Michał Bejger, Nelson Christensen, Thomas Dent, Philip Graff, Sergey Klimenko, Giulio Mazzolo, Atsushi Nishizawa, Florent Robinet, Patricia Schmidt, Rory Smith, John Veitch, Madeline Wade, Sofiane Aoudia, Sukanta Bose, Juan Calderon Bustillo, Priscilla Canizares, Colin Capano, James Clark, Alberto Colla, Elena Cuoco, Carlos Da Silva Costa, Tito Dal Canton, Edgar Evangelista, Evan Goetz, Anuradha Gupta, Mark Hannam, David Keitel, Benjamin Lackey, Joshua Logue, Satyanarayan Mohapatra, Francesco Piergiovanni, Stephen Privitera, Reinhard Prix, Michael Pürrer, Virginia Re, Roberto Serafinelli, Leslie Wade, Linqing Wen, Karl Wette, John Whelan, C. Palomba, G. Prodi

**Affiliations:** 1 INFN, Sezione di Roma, 00185 Rome, Italy; 2 LIGO-California Institute of Technology, Pasadena, CA 91125 USA; 3 Nikhef, Science Park 105, 1098 XG Amsterdam, The Netherlands; 4 N. Copernicus Astronomical Center, Bartycka 18, 00-716 Warsaw, Poland; 5 Carleton College, Northfield, MN 55057 USA; 6 Albert-Einstein-Institut, Max-Planck-Institut für Gravitationsphysik, 30167 Hannover, Germany; 7 NASA Goddard Space Flight Center, Greenbelt, MD 20771 USA; 8 University of Florida, Gainesville, FL 32611 USA; 9 Leibniz Universitat Hannover, 30167 Hannover, Germany; 10 Kyoto University, 6068502 Kyoto, Japan; 11 LAL Orsay, Universite Paris Sud, 91898 Orsay, France; 12 Cardiff University School of Physics and Astronomy, Cardiff, CF243AA UK; 13 University of Birmingham, Edgbaston, B15 2TT UK; 14 University of Wisconsin-Milwaukee, Milwaukee, WI 53201 USA; 15 Albert-Einstein-Institut, Max-Planck-Institut für Gravitationsphysik, 14476 Golm, Germany; 16 Washington State University, Pullman, WA 99164 USA; 17 University of the Balearic Islands, Palma, Spain; 18 Institute of Astronomy, Madingley Road, Cambridge, CB3 0HA UK; 19 University of Maryland, College Park, MD 20742 USA; 20 University of Massachusetts-Amherst, Amherst, MA 01003 USA; 21 University of Rome “Sapienza”, 00185 Rome, Italy; 22 European Gravitational Observatory (EGO), 56021 Cascina, Pisa Italy; 23 Instituto Nacional de Pesquisas Espaciais (INPE), Avenida dos Astronautas 1758, 12227-010 Sao Jose dos Campos, Brazil; 24 Tata Institute for Fundamental Research, Homi Bhabha Road, Colaba, Mumbai, 400005 India; 25 Department of Physics, Princeton University, Jadwin Hall, Princeton, 08544 NJ USA; 26 School of Physics and Astronomy, University of Glasgow, G12 8QQ Glasgow, UK; 27 Syracuse University, Syracuse, NY 13244 USA; 28 Universita’ di Roma Tor Vergata and INFN sezione di Roma Tor Vergata, 00133 Rome, Italy; 29 University of Western Australia, Crawley, Perth 6009 Australia; 30 Rochester Institute of Technology, Rochester, NY 14623 USA; 31 INFN, Gruppo Collegato di Trento, 38050 Povo, Trento, Italy; 32 Universit‘a di Trento, 38050 Povo, Trento, Italy

**Keywords:** Gravitational waves, Parameter estimation, Tests of general relativity, Compact binary merger, Neutron stars, Stochastic background, 60G35, 62F99, 62F15, 62M05

## Abstract

The Amaldi 10 Parallel Session C2 on gravitational wave (GW) search results, data analysis and parameter estimation included three lively sessions of lectures by 13 presenters, and 34 posters. The talks and posters covered a huge range of material, including results and analysis techniques for ground-based GW detectors, targeting anticipated signals from different astrophysical sources: compact binary inspiral, merger and ringdown; GW bursts from intermediate mass binary black hole mergers, cosmic string cusps, core-collapse supernovae, and other unmodeled sources; continuous waves from spinning neutron stars; and a stochastic GW background. There was considerable emphasis on Bayesian techniques for estimating the parameters of coalescing compact binary systems from the gravitational waveforms extracted from the data from the advanced detector network. This included methods to distinguish deviations of the signals from what is expected in the context of General Relativity.

## Introduction

The direct observation of gravitational waves (GWs) from astrophysical sources will be a major milestone in physics and astrophysics. The study of the detected waveforms will be a unique window into astrophysical sources as sites of strong-field and highly dynamical gravity. They will provide powerful insights into general relativity (GR) and perhaps into fundamental physics beyond GR.

These considerations have driven the development of kilometer-scale gravitational wave detectors. The Initial LIGO [1] and Virgo [2] detectors operated in the last decade with unprecedented precision, but no GW signals were detected.

The advanced detectors, Advanced LIGO [3] in the United States, Advanced Virgo [4] in Italy, and KAGRA [5] in Japan, will begin observing the sky in the next few years. The GW community fully expects to detect and study GWs with these detectors.

In parallel, techniques for detecting and analyzing the detected waveforms have been under development for more than two decades [6]. Many of these methods have been employed to search for GWs in Initial LIGO and Virgo data, which are dominated by instrumental noise and which contain significant non-Gaussian and non-stationary noise fluctuations. Recent efforts have focused on optimal and statistically rigorous extraction of astrophysical parameters from detected waveforms, and tests of General Relativity with these detections.

The Amaldi 10 Parallel Session C2 on GW search results, data analysis and parameter estimation included three lively sessions of lectures by 13 presenters, and over 30 posters. The talks and posters covered a huge range of material, including results and analysis techniques for ground-based GW detectors, targeting anticipated signals from different astrophysical sources: compact binary inspiral, merger and ringdown; GW bursts from intermediate mass binary black hole mergers, cosmic string cusps, core-collapse supernovae, and other unmodeled sources; continuous waves from spinning neutron stars; and a stochastic GW background.

We heard a comprehensive status report on searches for continuous waves from rotating neutron stars, including several innovative analyses in progress (Sect. 2.1). There was an overview of current and future searches for cosmological and astrophysical stochastic background signals, including a warning about correlated instrumental noise between detector sites in the network due to global Schumann resonances (Sect. 2.7).

There were presentations on results from LIGO and Virgo on searches for binary black hole mergers, using matched filtering techniques to target systems with total mass in the range of tens of solar masses (Sect. 2.6), and unmodeled burst techniques for systems of up to 1,000 solar masses, using anticipated data from the advanced detectors (Sect. 2.2). We also heard about a search for GW bursts from cosmic string cusps (Sect. 2.4).

It is challenging to apply matched filtering techniques to cover the large parameter space of possible signals from binary coalescence, especially in the presence of component spin, for which two component masses and two component spin vectors, or eight “intrinsic” parameters, are required. If the spins are not aligned with the orbital angular momentum, the orbital plane will precess about the total angular momentum axis, inducing significant amplitude and frequency modulation of the observed waveform. We heard about ongoing efforts to simplify the parameterization of spinning binary coalescence waveforms by capturing the effects of precession (Sect. 2.5).

There was considerable emphasis on Bayesian techniques for estimating the parameters of coalescing compact binary systems from the gravitational waveforms extracted from noisy detector data (Sect. 2.9). The effect of merger and ringdown on binary inspiral parameter estimation was discussed (Sect. 2.10). Estimating parameters in the presence of spin and precession is especially computationally expensive; we heard about the application of reduced order modelling to greatly speed up the computation of the waveforms (Sect. 2.13). Also presented were methods for using data from the coherent detector network to extract unmodeled transient burst waveforms from sources such as high mass binary coalescences or core-collapse supernovae (Sect. 2.3).

Parameter estimation is increasingly applied to help distinguish deviations of the GW waveform signals from what is expected in the context of General Relativity. In the case of binary inspiral waveforms, beyond-GR effects can be searched for using binary inspiral waveforms (Sects. 2.11, 2.12). The effects of a massive graviton with non-GR polarizations can be detected in the stochastic GW background (Sect. 2.8).

The analysis techniques continue to grow in scope, sophistication, power, variety and generality, in preparation for the advanced detector network. It was evident from both the talks and the many relevant posters that the GW community is transitioning from the setting of upper limits using initial LIGO and Virgo data, to preparations for discovery and detailed study of GW signals with data from the advanced detectors. Attendees got the strong sense that the community is ready for those data and those discoveries, and are eager to extract as much physics and astrophysics from the observations as possible. This made for a very stimulating and forward-looking session!

In Sect. 2, the authors provide summaries of their presentations, in the order that they were given in Session C2. Section 3 gives brief abstracts of the 34 posters that were submitted to the Session.

## C2 parallel session lecture presentations

### Searching for continuous gravitational wave signals from rotating neutron stars with the LIGO and Virgo detectors

Presented by M. Bejger, for the LIGO Scientific Collaboration and the Virgo Collaboration

Rotating neutron stars that emit continuous gravitational waves are among the most promising targets of the LIGO and Virgo detectors: sufficiently large non-axisymmetric deformation with respect to the axis of rotation of a star generates a time-varying mass-quadrupole moment and gravitational wave emission. The departure from an axisymmetric shape may be caused by the internal magnetic field and/or elastic stresses in the crust or core—if detected, it will provide an interesting insight into the presently obscured details of the interior neutron-star structure. The presentation summarized basic types of searches for such signals: *targeted* searches from known pulsars; *directed* searches from known locations on the sky; and *all-sky* wide parameter searches for unknown objects. Statistical methods used in the data analysis and to calculate the upper limits on the gravitational waves in the initial phase of LIGO and Virgo projects were briefly described. The presentation of recent results include beating the spin-down limit for the Crab and Vela pulsars [8, 10]; the search for a coherent signal from the direction of the Cas A supernova remnant [7], as well as Sco-X1 binary, SN1987A and the Galactic center with the cross-correlation method [9]; and the all-sky searches for signals of unknown position and frequency, with the use of the Poweflux [11] and Einstein@Home [12] pipelines.

### Perspectives for intermediate mass black hole searches with networks of second-generation ground-based gravitational-wave detectors

Presented by Giulio Mazzolo, in collaboration with F. Salemi, M. Drago, V. Necula, C. Pankow, G. A. Prodi, V. Re, V. Tewari, G. Vedovato, I. Yakushin and S. Klimenko

Strong evidence for the existence of intermediate mass black holes (IMBH, masses from ${\sim } 10^2$ to ${\sim } 10^5 \ \text{ M }_{\odot }$) is still lacking [13]. Compelling proof would be provided by the detection of GWs from coalescing IMBH binaries (IMBHB) [14, 15]. The sensitivity achieved by the GW detectors operating in the past years is insufficient to challenge the expected IMBHB merger rates [16]. However, in a few years, the second-generation (2G) interferometric detectors will come online [17]. The new class of instruments will consist of the upgraded LIGO and Virgo observatories and the KAGRA detector [3–5]. Compared to the previous detectors, the 2G observatories will share significantly higher sensitivity over a broader frequency band. We conducted simulation studies to assess the sensitivity of networks of 2G detectors to coalescing, non-spinning IMBHs. Waveforms modeling the gravitational radiation emitted by merging IMBHBs are added to 2G-detector simulated noise and searched for with the coherent WaveBurst data-analysis pipeline [18]. Results are presented for source-frame total masses between $50$ and $1{,}050 \ \text{ M }_{\odot }$ and mass ratios from 1:6 to 1:1. We find that the 2G observatories could be sensitive to the tested binary systems up to the Gpc range. A theoretical model is applied to estimate the IMBHB observation rate which might be achieved with the future instruments, yielding up to a few tens of events per year.

### Inverse problem for gravitational wave transients

Presented by S. Klimenko

The inverse problem—reconstruction of GW events and their parameters from signals recorded by detectors—is central in GW data analysis. Usually it is divided in two parts: separation of GW signals from the detector noise, and estimation of the signal parameters. This results in various detection statistics and methods for the source reconstruction. However, in general, these two parts should be considered together within the same analysis framework, called coherent network analysis, which combines data from all detectors. In this talk I describe the coherent network analysis in application to detection of poorly modeled transient GW signals. It employs the sparse time-frequency representation of GW data, dual stream analysis and constraints, which address the ambiguity of the inverse problem. Also I discuss the existing/future GW detector networks and their capabilities for detection, sky localization and reconstruction of source parameters.

### Searching for cosmic strings with the LIGO-Virgo gravitational-wave experiments

Presented by Florent Robinet for the LIGO Scientific Collaboration and the Virgo Collaboration

Cosmic strings are linear topological defects which are expected to form during symmetry-breaking phase transitions in the early universe [19]. Some inflation models based on string theory predict that fundamental strings and D-strings could grow to cosmic scales and constitute a network of cosmic superstrings [20]. When forming loops, cosmic strings radiate energy through bursts of gravitational waves in the presence of cuspy features [21]. This mechanism represents one of the most promising observational signatures to detect the existence of cosmic strings.

We searched for bursts of gravitational waves produced by cosmic string cusps in 625 days of data from ground-based interferometers LIGO and Virgo. No evidence for such events was found. However, it is possible to significantly constrain parameters of cosmic string models. Following the methods in [22], we constrained cosmic string networks described by three parameters: the string tension $G\mu$, the loop size parameter $\varepsilon$ and the reconnection probability $p$. This result [23] improves and complements existing limits from searches for a stochastic background of gravitational waves using cosmic microwave background and pulsar timing data. In particular, if the size of the loops is given by gravitational back-reaction, we place upper limits on the string tension $G\mu$ below $10^{-8}$ in some regions of the parameter space.

### Untangling precession to produce generic waveform models

Presented by Patricia Schmidt, in collaboration with Mark Hannam

One of the greatest theoretical challenges in the build-up to the Advanced GW detector era is the modeling of complete generic waveforms, in particular of precessing compact binaries. Precession occurs when the spin angular momenta of the individual holes are misaligned with the binarys orbital angular momentum, causing the orbital plane of the binary, as well as the spins, to precess. The time-dependent motion of the orbital plane is directly reflected in the emitted GWs in the form of phase and amplitude modulations.

In recent work we have introduced an approximation that has significantly simplified the problem of modeling precessing signals: we showed that generic precessing-binary inspiral waveforms, covering the full seven-dimensional parameter space, can be mapped to the two-dimensional subspace of non- precessing binaries, characterized by the mass ratio and a single effective total spin. As opposed to earlier modeling attempts, we identified the inspiral rate as the one of a very particular spin-aligned system via analyzing the waveforms in a co-precessing frame [26, 27], determined by maximizing the dominant harmonic. The mapping between precessing and non-precessing binaries then simply consists of three time-dependent rotations applied to the precessing waveforms. We found that this identification is extremely accurate, yielding matches $\ge$0.99 with parameter biases in the effective total spin of less than $0.02$. We concluded that the inspiral and precession dynamics decouple approximately. In order to utilize this approach to systematically model precessing binaries, we applied the inverse rotations to the corresponding non-precessing waveforms. We demonstrated the efficacy of this approach for pure post-Newtonian inspirals as well as for complete hybrid waveforms, obtaining matches $\ge$0.97 for most binary orientations [24]. This led to the proposal of the following general strategy to produce precessing waveforms, to “twist-up” corresponding non-precessing waveforms by applying the appropriate time-dependent rotations to them, as outlined in [24] [see Eq. (5.4)]. Additionally, we showed evidence that the leading-order precession effects can be efficiently modeled with a small number of parameters [28]. This “twisting-up” approach has recently lead to the construction of a complete phenomenological inspiral-merger-ringdown waveform model in the frequency domain [25] as well as to a precessing effective-one-body model [29]. Here, we summarized the individual components of the modeling process, discussed the modeling of precession effects with a single precession parameter and briefly presented the complete, phenomenological frequency-domain inspiral-merger-ringdown waveform model for precessing binary-black-holes, PhenomP.

### Searching for stellar-mass binary black hole coalescences with ground-based interferometers

Presented by Thomas Dent for the LIGO Scientific Collaboration and the Virgo Collaboration

Binary black holes with components in the 3–50 solar mass range are potentially the brightest GW sources in their sensitive frequency band. Both the properties of these binaries (masses and spins) and their coalescence rates are subject to large uncertainties; thus positive detections, or even stringent rate limits, could provide unique information that would allow us to select between models of binary formation and evolution.

I present the results of searches for these binaries in the most recent joint LSC-Virgo science run [30, 31]. The maximum sensitive distance of the detectors over this period for a (20, 20) solar mass coalescence was 300 Mpc. No gravitational-wave signals were detected. We find 90 %-confidence upper limits on the coalescence rates of binary black hole systems with non-spinning components as follows: for systems with components of $5 \pm 1$ solar masses, a limit of $6.4 \times 10^{-6} /\hbox {Mpc}^3/\hbox {year}$; for systems with components between 19 and 28 solar masses, a limit of $3.3 \times 10^{-7}\, /\hbox {Mpc}^3/\hbox {year}$.

I also discuss the science case and prospects for detection in the Advanced detector era, and technical challenges faced by current and future searches.

### Stochastic gravitational wave background searches with advanced LIGO and advanced Virgo: strategies and goals

Presented by Nelson Christensen for the LIGO Scientific Collaboration and the Virgo Collaboration

The Advanced LIGO [3] and Advanced Virgo [4] detectors are expected to start acquiring data in 2015, and work toward their target sensitivity over the subsequent years. A major goal for LIGO and Virgo will be to detect or set limits on the energy density of a stochastic background of gravitational waves. A stochastic background of gravitational waves is expected to arise from a superposition of a large number of unresolved cosmological and/or astrophysical sources. A cosmologically produced background would carry unique signatures from the earliest epochs in the evolution of the Universe. Similarly, an astrophysical background would provide information about the sources that generated it. Advanced LIGO and Advanced Virgo observations should be able to probe interesting regions of parameter space for these models. LIGO and Virgo’s search strategies for these signals were reported in the talk.

Using LIGO science run S5 data, an upper limit on the energy density of gravitational waves (from 41 to 169 Hz) was set to be $\varOmega _{GW} < 6.9 \times 10^{-6}$ [32]. As Advanced LIGO and Advanced Virgo come online and work through commissioning to achieve their target sensitivities, there will be a few data collection science runs [33]. With 70 days of early commissioning LIGO observations, an upper limit of $\varOmega _{GW} < 6.9 \times 10^{-7}$ can be achieved (15–200 Hz). During the mid-commissioning time period and with 6 months of observations, there could be an upper limit of $\varOmega _{GW} < 2.4 \times 10^{-8}$. A 9-month period of observation during the late commissioning era will give an upper limit of $\varOmega _{GW} < 5.0 \times 10^{-9}$. Finally, when the target sensitivities are achieved, a year long observation run will allow for an upper limit of $\varOmega _{GW} < 1.0 \times 10^{-9}$, while 3 years of observations will give an upper limit of $\varOmega _{GW} < 5.8 \times 10^{-10}$.

In addition, there was a discussion on how global electromagnetic noise (Schumann resonances) will affect the LIGO and Virgo search for a stochastic gravitational wave background, and possible strategies were presented on how to monitor and subtract this potential source of correlated noise in a the global detector network [34]. This correlated noise could potentially be a problem for LIGO and Virgo as they strive to set these stringent upper limits or make a detection.

### Probing for massive stochastic gravitational-wave background with a detector network

Presented by Atsushi Nishizawa

In a general metric theory of gravitation in four dimensions, six polarizations of a gravitational wave are allowed: two scalar and two vector modes, in addition to the two tensor modes predicted in general relativity. Also graviton mass, which could be different in each polarization, is another characteristic of modification of gravity. Thus, testing the existence of additional polarization modes and graviton mass can be a model-independent test of gravity theories. In this presentation, we have studied the search method for a massive stochastic gravitational-wave background (GWB) with tensor, vector, and scalar polarization modes, extending the ordinary method for massless graviton. If a GWB is massive, the phase velocity, which is larger than the speed of light, affects the cross-correlation statistics. A GWB spectrum has a lower frequency cutoff corresponding to the graviton mass. Using the Fisher information matrix, we have investigated the detectability of graviton mass with ground-based detectors. We find that if a GWB is detected at the level of $h_0^2 \varOmega _{\mathrm{{gw}},0}=10^{-7}$, we can determine the mass of graviton in the range of $7\times 10^{-15}\,\mathrm{{eV}} < m_g < 2\times 10^{-13}\,\mathrm{{eV}}$ for each polarization mode. We also showed that even if the GWB signal is a mixture of three polarization modes, we can safely separate them and determine the mass of the graviton. Even if a GWB is detected but the lower frequency cutoff is not detected, we can set an upper limit on graviton mass, which is the lower cutoff of detector noise curve, say, ${\sim } 10\,\mathrm{{Hz}}$ or ${\sim } 7\times 10^{-15}\,\mathrm{{eV}}$.

### Parameter estimation for compact binary coalescence signals with LIGO-Virgo

Presented by John Veitch in collaboration with Vivien Raymond, for the LIGO Scientific Collaboration and the Virgo Collaboration

The signals emitted by coalescence of compact binaries (black holes and neutron stars) will encode details of the sources that are uniquely measurable with gravitational waves, including the masses of the compact objects, their spins, position and distance. However, the accuracy of such inferences is limited by the detector noise (including unmodeled artefacts present in the data), uncertainties in the waveform models, and in the detector calibration.

Recent progress has been made in the development of robust and efficient methods of solving the parameter estimation problem for compact binaries [35]. These make use of probabilistic Bayesian methods (MCMC and nested sampling) to estimate the posterior probability distribution for the signal parameters. These methods were applied to data collected during the most recent joint science run of the LIGO and Virgo detectors, which contained a range of hardware and software injections, including a “blind injection” where the presence of the simulated signal was initially hidden from the collaboration [30]. The results of these simulations demonstrate that we are able to recover the source parameters for systems which span the neutron-star and black-hole binary parameter space between 1 and 25 $M_\odot$, and include spins on both bodies in the analyzed models. Further development of the analysis methods will focus on improving computational efficiency and robustness against unmodeled noise fluctuations, in preparation for the advanced detector era and the first detected signals.

### Effect of merger and ringdown signals on the estimation of parameters of binary black holes

Presented by Philip Graff, in collaboration with Alessandra Buonanno and B.S. Sathyaprakash

In this study, we perform a Bayesian analysis of massive binary black hole systems using effective-one-body waveforms. Our waveform model includes merger and quasi-normal modes (QNMs) that are tuned to numerical relativity results for many spherical modes of radiation. The additional modes help determine the parameters of progenitor binaries even when their inspiral phase might not be in the sensitivity band of a detector. These analyses used the advanced LIGO zero-detuned high-power (ZDHP) noise power spectrum with a lower frequency cut-off of $10\, \mathrm Hz$. As the binary’s total mass increases, less of the inspiral phase is in the sensitive band of the detector, leading more of the information to be contained in the merger and ringdown QNMs. As these depend on the final mass of the black hole as opposed to the progenitor masses ($m_{1,2}$), the total mass of the system will become more precisely measured than the chirp mass (a function of $m_{1,2}$ that is primary in describing the phase evolution during inspiral) above a mass of ${\sim } 110 \mathrm M _\odot$.

By including sub-dominant spherical modes beyond the dominant $(2,2)$ mode in our waveform template model, we are able to reduce both statistical bias and uncertainty in the measurement of the binary’s masses. The relative amplitudes of the modes provide important information about the mass ratio of the binary system. Furthermore, this extra information helps to break degeneracies and allow for measurement of extrinsic parameters that will be important for astronomical follow-up and study (mainly sky location and luminosity distance). In this regime, the large uncertainties make clear the importance of the choice of prior probability distributions: different priors will introduce different biases to the final measurements for signals just above the detection threshold.

### TIGER: a data analysis pipeline for testing GR with gravitational waves from coalescing binaries

Presented by Michalis Agathos, in collaboration with T. G. F. Li, W. Del Pozzo, C. Van Den Broeck, J. Veitch, and S. Vitale

The detection of GW signals from coalescing compact binary systems will, for the first time, give us access to the relativistic, strong-field dynamics of gravity. Given a set of GW signals, one of the most important questions is that of their compatibility with the predictions of general relativity. We have developed TIGER, a Bayesian data analysis pipeline for testing general relativity using GW signals from binary neutron stars, first introduced by Li et al. [36–38], where a proof of principle, as well as performance under a few non-trivial scenarios were demonstrated (for a review, see [39]). This framework does not rely on any particular alternative theory of gravity, is well-suited to low signal-to-noise detections, and will allow us to combine information from multiple detections in a straightforward manner. The TIGER pipeline is mature for the case of binary neutron stars, and in this study [40] we test its robustness against a number of nuisance effects of fundamental, astrophysical or instrumental nature. In particular we address the following issues one by one: What would be the effect of precessing spins? Can neutron star tidal effects of unknown magnitude be separated from GR violations? How does the pipeline treat simulated signals generated by different approximants and, by extension, a real signal? How would the truncation of the post-Newtonian expansion of the phase affect the performance of the pipeline? What would be the effect of detector calibration errors? It was shown that none of these issues will impede our ability to test GR.

### Advanced LIGO’s ability to detect apparent violations of the cosmic censorship conjecture and the no-hair theorem through compact binary coalescence detections

Presented by Madeline Wade, in collaboration with Jolien D.E. Creighton, Evan Ochsner, Alex B. Nielsen

In anticipation of the new era of gravitational wave detectors, it is especially important to develop methods for gaining information about astrophysical systems from gravitational wave signals. We studied a method for testing the cosmic censorship conjecture and the no-hair theorem using the inspiral portion of the compact binary coalescence gravitational waveform. The cosmic censorship conjecture implies a limit on the spin to mass squared ratio of a black hole. The no-hair theorem implies that a black hole should not be tidally deformed. We studied Advanced LIGOs ability to detect violations of the cosmic censorship conjecture and the no-hair theorem in the Kerr geometry. We used the Fisher information matrix to calculate the measurability of the spin, mass, and tidal parameters appearing in the gravitational waveform, and then determined if the measurability of these parameters will allow for the detection of a violation of either cosmic censorship or the no-hair theorem. We investigated two ways to improve the measurability of relevant parameters, and therefore improve our ability to test conjectures in general relativity. First, we studied the importance of a physical prior on the symmetric mass ratio for measuring spin and tidal parameters. We found that a physical prior on the symmetric mass ratio can lead to improved measurability of spin when using a Newtonian amplitude waveform for certain astrophysical systems. We also studied how higher harmonics included in the gravitational waveform would affect the measurability of relevant parameters. Higher harmonics can lead to parameter degeneracy breaking, which results in greatly improved parameter measurability of both spin and tides for certain astrophysical systems. These effects are most important for near-equal mass binary black hole systems.

### Towards rapid parameter estimation on cbc sources with advanced detectors

Presented by Rory Smith, in collaboration with Chad Hanna, Ilya Mandel and Alberto Vecchio

Estimating the parameters of coalescing compact binaries in a full Bayesian framework is essential to gravitational-wave astronomy, but can be highly computationally intensive, with analyses on a single stretch of data taking up to several hundred hours. Here we showcase two techniques which may enable low latency (Bayesian) parameter estimation in the advanced detector era. These are: (1) using interpolated template gravitational waveforms as filters of the data [41], and (2) directly interpolating the compact binary likelihood function [42]. Both techniques have the potential to reduce the computational cost of parameter estimation by at least an order of magnitude. Our results have important implications for both parameter estimation and coherent searches for gravitational-waves from compact binary sources, and we discuss these in the context of advanced LIGO.

## C2 parallel session poster submissions

*Progress of the low latency pipeline for the gravitational wave detector Mario Schenberg. By Da Silva Costa C., Denis Aguiar O., Fauth A.* The Mario Schenberg gravitational wave antenna, located in Sao Paulo, is a spherical resonant mass detector, which is 65cm in diameter and weighs 1,150 kg. The antenna is currently being upgraded and the next run will occur later this year. Since the detector last operated in 2008, its 6 parametric transducers, measuring the sphere quadrupolar mode oscillations, have been redesigned to increase their mechanical Q. Spherical detectors present the ability to determine simultaneously both source direction and signal polarization. We have developed a low latency pipeline that takes advantage of these abilities and processes real time data in less than one-sixth the acquisition time. For each reconstructed direction, its resolution and systematic error are determined using a mapping error system. To reduce the false alarm rate, triggers are vetoed using information from the spherical modes. In the near future, we expect to add a matched filter to the pipeline. This will increase GW parameter resolution and reduce the false alarm rate. As a future part of the pipeline, a cosmic ray veto was added to the Mario Schenberg setup in December 2011. Since then, it is acquiring data and we are analyzing the local multiplicity. The energy deposition due to cosmic rays on the sphere is presently being simulated.

*Inspiral Dynamics of Spin Dominant Compact Binaries. By Gopakumar A., Gupta A. * We follow the secular evolution of unequal mass spinning compact binaries whose dominant spin angular momentum exceeds the orbital counterpart at $f_0$, the initial frequency of an interferometric detector like Advanced LIGO. These binaries can experience transitional precession before their GW frequencies cross $f_0$, provided their essentially constant dominant spin-orbit misalignments are ${>}170^{\circ }$. The usual inspiral templates turn out to be not very faithful in modeling the associated GWs, though the precessional dynamics are simple when these binaries inspiral through $f_0$. A prescription is presented to model the GWs from the inspiral phase of such binaries in a faithful manner. Additionally, we explore the effect of gravitational radiation reaction on the orientation of the dominant spin from the initial direction of the total angular momentum $\mathbf{j}_0$. The dominant spin orientations from $\mathbf{j}_0$ are ${<}45^{\circ }$ at $f_0$ for binaries with dominant spin-orbit misalignments ${<}170^{\circ }$ and rather unpredictable for higher dominant spin-orbit misalignments.

*Studying the effects of tidal corrections on parameter estimation. By Wade L.* Tidal deformations of neutron stars in binary systems during the infall of gravitationally radiating neutron stars before merger call for analytic corrections to the post-Newtonian gravitational-wave waveform. Tidal deformation information is important to searches for gravitational waves from these sources because it can break degeneracies in the estimation of the physical parameters of the binary, and it could also lead to insights about the neutron star equation of state. Using full Bayesian MCMC (Markov Chain Monte Carlo) simulations, we studied how tidal corrections affect and inform parameter estimation for binary neutron stars. We also investigate the systematic biases that arise in parameter estimation from using different post-Newtonian waveform families.

*Measuring the neutron-star equation of state with multiple binary neutron star inspiral events. By Lackey B.* Gravitational waves from compact binaries containing neutron stars can provide useful constraints on the neutron-star equation of state. In binary neutron star systems this information comes mainly from the imprint of the neutron-star tidal deformability on the waveform during inspiral. Previous work has shown that the tidal deformability is measurable with Advanced LIGO for a single event when including the tidal effect up to merger. In this work we describe a method for stacking measurements of the tidal deformability from multiple inspiral events to measure the unknown parameters of a parametrized equation of state. Specifically, we use a 4-parameter piecewise polytrope that matches theoretical equation of state models to a few percent to determine the accuracy that one can measure the pressure at twice nuclear density as well as the adiabatic index in three density regions. We also examine how the uncertainties in the equation of state parameters depend on the number of observations and on the distribution of neutron-star masses in binary neutron star systems.

*Effect of the higher order modes of GWs emitted from binary black hole mergers measured by a GW burst search algorithm. By Mohapatra S., Clark J., Cadonati L. * The GWs emitted from the merger of binary black holes can be expressed as the dominant quadrupole modes and sub-dominant higher order modes, in the spin-weighted spherical harmonics basis. Until now all the gravitational-wave searches that were conducted have utilized only the signal morphology of the dominant quadrupole modes for the interpretation of the search results. It is qualitatively known that these sub-dominant modes can be crucial for certain source orientations and source parameters of the binary black holes. Recently, an analytical family of binary black hole GW signals including higher order modes became available. We present a study quantifying the effect of the sub-dominant modes on the expected signal-to-noise ratio measured by a morphology-independent gravitational-wave burst search.

*Inferring core-collapse supernova physics with GWs. By Logue J.* Stellar collapse and the subsequent development of a core-collapse supernova explosion emit bursts of GWs that might be detected by the advanced generation of laser interferometer gravitational wave observatories such as Advanced LIGO, Advanced Virgo, and KAGRA. GW bursts from core-collapse supernovae encode information on the intricate multi-dimensional dynamics at work at the core of a dying massive star and may provide direct evidence for the yet uncertain mechanism driving supernovae in massive stars. Recent multi-dimensional simulations of core-collapse supernovae exploding via the neutrino, magneto-rotational, and acoustic explosion mechanisms have predicted GW signals which have distinct structure in both the time and frequency domains. Motivated by this, we describe a promising method for determining the most likely explosion mechanism underlying a hypothetical GW signal, based on Principal Component Analysis and Bayesian model selection. Using simulated Advanced LIGO noise and assuming a single detector and linear waveform polarization for simplicity, we demonstrate that our method can distinguish magneto-rotational explosions throughout the Milky Way (10 kpc) and explosions driven by the neutrino and acoustic mechanisms to 2 kpc. Furthermore, we show that we can differentiate between models for rotating accretion-induced collapse of massive white dwarfs and models of rotating iron core collapse with high reliability out to several kpc.

*A gravitational-wave search algorithm for non-precessing spinning binary black holes. By Privitera S., Mohapatra S., Hanna C., Fotpolous N., Ajith P., Weinstein A., Whelan J.* Previous searches for gravitational waves in LIGO-Virgo data from the inspiral, merger and ringdown of binary black holes have used a matched-filter approach with non-spinning templates. However, astrophysical black holes in binaries are expected to have significant spin; neglecting such effects in the templates may reduce the detection efficiency. Gravitational-wave signal models from the coalescences of non-precessing spinning binary black holes have recently become available, but it is not known whether using these templates in a search can improve detection efficiency of generic-spinning binary black holes signals in realistic, non-Gaussian noise from the Initial LIGO detectors. We present [43] a search method for gravitational-waves that employs non-precessing spinning templates and compare the performance of this method to one in which the templates neglect spin effects.

*Science reach of stochastic gravitational wave background searches with second-generation detectors. By Mandic V.* The stochastic GW background is produced as an incoherent superposition of gravitational waves from many cosmological and astrophysical sources, and could therefore carry unique information about cosmological and astrophysical processes that gave rise to it. I present a new formalism designed to extract this information from the stochastic background measurements. The formalism offers the prospect of estimating the energy budget of the stochastic gravitational wave background, and provides a natural framework for inclusion of other measurements to further constrain model parameters. I discuss applications of this formalism to some specific situations, for example, to measure possible parity violation in the early universe, or to probe the stochastic background models based on coalescences of compact binaries.

*Inferences from the post-merger gravitational wave signal in binary neutron star coalescence. By Clark J.* The inspiral phase of binary neutron star coalescence is widely considered to be one of the strongest sources in the next generation of ground based gravitational wave detectors, yielding detection rates in the range of 0.4–400 events per year of operation. Amongst the most exciting prospects for gravitational wave astronomy is the measurement of neutron star mass and radius, which would lead to constraints on the neutron star equation of state. Recent numerical simulations suggest that, rather than prompt collapse to a black hole, the favored outcome of the merger is the formation of a relatively long-lived hyper-massive neutron star whose gravitational wave signal constitutes a significantly weaker but complementary gravitational wave source to the inspiral phase. We present results of deploying a Bayesian nested sampling algorithm in the context of a gravitational wave inspiral-triggered search for, and characterization of, this post-merger signal.

*Impact of higher harmonics in searching for gravitational waves from binary black hole coalescence in advanced LIGO. By Capuano C.* Current searches for gravitational waves from compact binary coalescence use dominant- mode only waveforms as templates in a matched filter. It has been shown that neglecting additional modes causes mismatch between these templates and expected signals. We investigate the effect of this mismatch on signal-based vetoes and the detection efficiency of advanced LIGO detectors for non-spinning stellar-mass binary black holes. We also consider what improvement could be expected if a search were developed that utilized these additional modes.

*Gravitational-wave parameter estimation with compressed likelihood evaluations. By Canizares P., Field S., Gair J., Tiglio M.* One of the main bottlenecks in GW astronomy is the high cost of performing parameter estimation and GW searches on the fly. We propose a novel technique based on Reduced Order Quadratures (ROQs), an application and data-specific quadrature rule, to perform fast and accurate likelihood evaluations. These are the dominant cost in Markov chain Monte Carlo (MCMC) algorithms, which are widely employed in parameter estimation studies, and so ROQs offer a new way to accelerate GW parameter estimation. We illustrate our approach using a four dimensional GW burst model embedded in noise. We build an ROQ for this model, and perform four dimensional MCMC searches with both the standard and ROQs quadrature rules, showing that, for this model, the ROQ approach is around 25 times faster than the standard approach with essentially no loss of accuracy. The speed-up from using ROQs is expected to increase for more complex GW signal models and therefore has significant potential to accelerate parameter estimation of GW sources such as compact binary coalescences.

*Methods for the first all-sky search for continuous gravitational waves from spinning neutron stars in binary systems. By Goetz E.* An all-sky search for continuous gravitational waves from unknown neutron stars in binary systems is daunting in its computational challenge, because one much search over additional binary orbital parameters. A new search algorithm, called TwoSpect, has been developed and implemented; it exploits the periodic orbital modulation of the source waves by searching for patterns in doubly Fourier transformed data. This technique enables a more computationally efficient search compared to other StackSlide-like, all-sky search algorithms. We present the analysis methods and current status of the first ongoing search for sources in binary systems in the LIGO Science Run 6 and Virgo Science Runs 2 and 3 data sets.

*Resolving noise structures in Virgo and LIGO data: an application of non-linear system identification. By Piergiovanni F., Guidi G., Carini A.* A gravitational wave detector features many kinds of non-linear physical processes. Disturbances present in auxiliary channels, including narrow spectral features, may be converted linearly and non-linearly into noise, polluting the gravitational channel in a wide frequency range. Uncovering such relationships between auxiliary channels and the gravitational wave channel can be very useful for characterizing the detector and possibly also for improving the confidence of gravitational wave searches. We present a system identification tool developed to characterize linear and non-linear noise in the gravitational wave detector output. The signal is expanded in a Volterra series of the auxiliary channels, and the model parameters are determined by means of the Sorted Fast Orthogonal Search technique. Cross-correlation is used to perform dephasing identification, to minimize the number of time lags in Volterra expansions. The magnitude of the specific contribution of any channel or combination of channels is estimated; this allows for the recognition of the channels that are most involved in the noise structures, giving hints about noise sources. We applied the tool to interferometric detector data and showed that it is effective in performing blind identification among several hundreds of auxiliary channels in linear and bilinear combination.

*Gravitational waves from SCO X-1 : prospects for detection and a comparison of methods. By Crowder G., Dergachev V., Galloway D., Goetz E., Meadors G., Messenger C., Premachandra S., Riles K., Sammut L., Thrane E., Whelan J.* The low-mass X-ray binary Scorpius X-1 is potentially our most luminous source of continuous gravitational radiation. Unlike for the recycled pulsars already targeted by LIGO-Virgo, this radiation would be powered by the accretion of matter from its binary companion rather than its rotational energy. With the advanced detector era fast approaching, work is underway to develop an array of robust tools for maximizing the science and detection potential of Sco X-1. It will be possible with advanced detector data to attain sensitivities below the current theoretical torque-balance limits, implying that signal detection is a possibility. We describe the plans and progress of a project designed to compare and contrast the numerous independent search algorithms currently employable. We describe a mock-data-challenge by which the search pipelines will test their relative proficiencies in parameter estimation, search volume dependence, computational efficiency, robustness, and most importantly, search sensitivity.

*Parameter-space metric for All-sky coherent searches for gravitational-wave pulsars. By Wette K., Prix R.* All-sky, broadband searches for gravitational-wave pulsars are computationally limited. It is therefore important to make efficient use of available computational resources, for example by minimizing the number of templates needed to cover the parameter space of sky position and frequency evolution. For searches over the sky, the required template resolution is different for each sky position. This makes it difficult to achieve an efficient covering. Previous work on this problem has found choices of sky and frequency coordinates, with respect to which the parameter space metric (which determines the template resolution) is constant. These approaches, however, are limited to coherent integration times of a few days, which in turn limits the sensitivity achievable by e.g. a hierarchical search pipeline. We present recent work on new sky and frequency coordinates, with a flat parameter space metric, that do not suffer from this limitation. By allowing integration times of, e.g., longer than a week, improvements in search sensitivity may be possible using the new coordinates.

*Effect of sine-gaussian glitches on searches for binary coalescence. By Dal Canton T., Bhagwat S., Dhurandhar S., Lundgren A.* We investigate the effect of an important class of glitches occurring in the detector data on matched filter searches of gravitational waves from coalescing compact binaries in the advanced detector era. The glitches, which can be modeled as sine-Gaussians, can produce triggers with significant time delays and thus have important bearing on veto procedures. We provide approximated analytical estimates of the trigger SNR and time as a function of the parameters describing the sine-Gaussian (center time, center frequency and Q-factor) and the inspiral waveform (chirp mass). We validate our analytical predictions through simple numerical simulations, performed by filtering noiseless sine-Gaussians with the inspiral matched filter and recovering the time and value of the maximum of the resulting SNR time series. Although we identify regions of the parameter space in which each approximation no longer reproduces the numerical results, the approximations complement each other and together effectively cover the whole parameter space.

*New Einstein@Home directed search for young compact objects. By Prix R.* Einstein@Home is beginning a new semi-coherent search on S6 data, targeting the sky-positions of about 20 young non-pulsing neutron stars (NS) or NS candidates in supernova remnants (including Cas-A and VelaJr). These objects may be detectable if they spin within the (best) LIGO band, and are slowing down due to gravitational waves, starting from a near-breakup rotation at birth. Non-detection would therefore allow us to set constraining upper limits. The sensitivity of this search is expected to improve by about a factor of 2 on previous upper limits set on these objects. Here we present some of the considerations that went into designing this search, addressing the following questions: What astrophysical priors can we use? What parameter space can we cover? How can we distribute computing power to obtain the best detection probability? How can we balance these aims with the practical constraints of an Einstein@Home search?

*An F-statistic based multi-detector veto for detector artifacts in continuous gravitational wave searches. By Keitel D., Prix R., Papa M.A., Leaci P., Siddiqi M.* The emission of continuous gravitational waves (CWs) is expected from spinning neutron stars with non-axisymmetric deformations. Detecting these very weak signals requires very sensitive instruments and data analysis techniques. The standard multi-detector F-statistic often used for CW data analysis is optimal in Gaussian noise, but susceptible to false alarms from noise artifacts in the form of strong monochromatic lines. In the past, ad-hoc post-processing vetoes have been used to remove these artifacts. Here we provide a systematic framework to derive a generalized form of such line vetoes (LVs). With an extended noise model including a hypothesis for single-detector lines, we can use a Bayesian odds ratio to derive a generalized detection statistic: the LV-statistic. Compared to the F-statistic, it requires very little extra computational effort. We test this LV-statistic on both simulated and real detector data from the first year of the fifth LIGO science run. We show that the LV-statistic retains most of the detection power of the F-statistic in Gaussian noise, while being much more robust in the presence of line artifacts. Furthermore, we briefly describe the advantages, in the context of Einstein@Home, of applying the LV-statistic directly on the host machines, as done in a recent search that analyzed data from the sixth LIGO science run, and whose post-processing is currently underway.

*Application of the 5-vector analysis method to narrow-band searches of continuous gravitational wave signals. By Serafinelli R., Astone P., Colla A., D’Antonio S., Frasca S., Palomba C.* Targeted searches of continuous waves from spinning neutron stars normally start with the assumption that the frequency of the gravitational wave signal is at a given known ratio with respect to the rotational frequency of the source, e.g. twice for an asymmetric neutron star rotating around a principal axis of inertia. In fact, this assumption may well be invalid if, for instance, the gravitational wave signal is due to a solid core rotating at a slightly different rate with respect to the star crust. Then, it is important to perform *narrow-band* searches in which a small frequency band (and frequency derivative range) around the electromagnetic-inferred values is explored. In order to implement this, we have adapted a computationally efficient analysis method based on 5-vectors, which was originally developed for targeted searches. In this work, the basic principles of this new procedure, together with results of tests done using Virgo VSR1 data, are discussed. We have also estimated the sensitivity of the method, on the base of which we expect a *narrow-band* search of 0.02 Hz around Crab pulsar central frequency, done over Virgo VSR4 data, could improve results of other similar searches and overcome the *spin-down limit* by about a factor of 2.

*Avoiding selection bias in gravitational wave astronomy. By Veitch J. , Messenger C.* Ground-based GW searches typically use a detection threshold to reduce the number of background triggers, but imposing such a threshold will also discard some real signals of low amplitude. This process can produce a selection bias in results drawn from the population of triggers unless the discarded data is properly accounted for. We will describe how selection bias can be naturally avoided by considering both the triggers and our ignorance of the sub- threshold triggers which are discarded. This approach produces unbiased estimates of population parameters even in the presence of false alarms and incomplete data.

*Low latency search for gravitational waves from compact binary coalescence. By Wen L.* For advanced gravitational-wave detectors, signals from coalescing binaries of neutron stars and stellar-mass black holes could be detected before or near their merger. I will discuss the astrophysical motivation for fast low-latency detection of such signals and present a recently developed time-domain search technique aiming at extremely low latency search for gravitational waves from binary coalescence. I show the result of the sensitivity our pipeline tested on simulated data as well as existing detector data and recent Engineering Run data that simulate online data from advanced gravitational-wave detectors. I’ll also present our on-going effort to improve the computational efficiency of the pipeline by using the cost-effective Graphics Processing Unit as well as a new template interpolation strategy. Implications for future joint gravitational wave and electromagnetic observations will be discussed.

*Running the frequency Hough all-sky continuous wave analysis on the grid: a job submission and control framework. By Colla A., Astone P., D’Antonio S., Palomba C., Frasca S.* In the all-sky search for continuous gravitational wave signals from unknown sources, one must apply a hierarchical approach in searching the source parameter space (source coordinates, frequency and frequency derivatives). The very high computational requirements of this search are addressed by most analysis pipelines, such as the one based on the Frequency Hough algorithm, by splitting the analysis in a series of parallel and independent tasks and running them in a distributed computing environment. This translates into tens of thousands of jobs which have to be configured, submitted and monitored. We will describe the software framework we have developed to automatize the submission, monitoring, failure recovery and output retrieval of the analysis jobs within the Grid environment. We are currently applying it to the Frequency Hough all-sky search using data from the Virgo second and fourth science runs, and we will report on how the framework helps to increase the overall efficiency of the analysis.

*Testing the validity of the single-spin approximation in IMR waveforms. By Pürrer M., Hannam M., Ajith P., Husa S.* An effective single spin parameter allows us to capture the dominant spin effects in coalescences of non-precessing compact binaries, while reducing the number of physical parameters in a waveform model. To leading order an optimal effective spin parameter is available in the PN regime and an inspiral model based on it has been shown to be effectual (and faithful when the masses or spins are equal). Recent phenomenological inspiral-merger-ringdown (IMR) models for black-hole binaries have used a similar parameter. We quantify how well the single spin approximation works for a set of BBH configurations with mass-ratio 4 and effective spin 0.45 in terms of parameter biases and uncertainties.

*Parameter estimation improvements using a new hybrid waveform. By Aoudia S., Babak S., Hinder I., Ohme F., Petiteau A., Sesana A., Wardell B.* Detection of gravitational waves by a space-based gravitational wave observatory such as eLISA-NGO requires not only an optimization over all the instrumental equipments but also an optimization of our knowledge about waveforms, especially to improve our ability to extract information on the physical parameters of the source. Our work aims to illustrate, indeed, the importance and power of including the full waveform modeling. Especially, it reinforces a recent study which shows that by comprising all the stages of the binary merger, it is possible to reach a high level of precision measurements. For this study we build a new hybrid model, resulting from a very accurate matching between PN and NR waveforms. The matching is done for several mass ratios (q = 1, 2, 3 and 4) by fixing spins and orbital angular momentum of the system. By the use of this new hybrid model, errors on parameters based on Fisher matrix was computed for several thousand sources by randomly choosing 7 free parameters among the 15 characterizing a coalescent binary. For the same sources the same work was repeated twice, using a pure PN model (i.e. including only the inspiral phase): (1) with 7 free parameters, and (2) with 15 free parameters. The comparison of all these results enables us to compute a new law which may be used to shift or improve the precision on the measurements coming from any more realistic catalogue where the Fisher matrices were only computed using a pure PN model.

*The noise characterization framework for Advanced Virgo detector. By Cuoco E., Hemming G., Berni F., Cortese S., Colla A., Drago M., Re V., Piergiovanni F., Guidi G., Vajente G.* We are approaching the advanced detector era and we have to be ready with useful tools to help the commissioning phase. We gained experience from the Virgo detector, understanding the importance of having tools to be used either for a fast noise characterization or for prompt reaction to mitigate the noise disturbances. We are working on the upgrade of the Noise Monitor Application Programming Interface (NMAPI), which gathers both in-time and on-line noise analysis pipelines. Our goal is to set up a framework in which spectral characterization is integrated with linear and non-linear noise coupling and with slow and fast non-stationarity tracking. Moreover we plan to give the users, either data analysis or commissioning people, a simple web interface to retrieve information from different pipelines. In this work, we describe the framework into which these tools are integrated, and examples of the frameworks implementation and applications.

*Calculating the significance of candidate binary coalescence signals. By Dent T.* In order to report detections of transient gravitational wave signals it is necessary to obtain high statistical confidence, i.e. low false alarm probability. Doing this in real data containing a population of loud, unmodeled non-Gaussian transients (‘glitches’) presents several technical challenges. The established method of background estimation is to apply unphysical time-shifts to detectors at different sites. However, this can be computationally expensive, requiring long stretches of data to establish high confidence, and prone to large statistical fluctuations. I will present the results of investigations into computationally efficient and reliable methods of finding the statistical significance of candidate events, including comparisons with existing methods, with a view to application on advanced detector data.

*Impact of noise cancellation on the search for gravitational wave transient signals. By Re V., Drago M., Klimenko S., Mazzolo G., Necula V., Prodi G., Salemi F., Tiwari V., Vedovato G., Yakushin I.* One of the prominent problems in the search for GW is the presence of non gaussian excess noise which may hide a GW signal due to increased false alarm rate. Regression analysis of auxiliary environmental and instrumental channels can provide a partial noise cancellation by measuring the linear (or non linear) coupling of the auxiliary channels to the GW channel, thus subtracting such predicted contributions from the noise. In this work we explore the impact that this method has on the search for transient GW signals. We present preliminary results of the application of noise cancellation on a set of real data from the first joint LIGO-Virgo run. The efficiency of the method and the impact on the search sensitivity is tested by means of software injections simulating transient signals in the data. Investigations are being pursued in particular within the lower frequency band (${<}100$ Hz ) which will be of particular astrophysical interest in future observations by the advanced detectors, being crucial for the achievable range of BH–BH coalescences.

*Searching for a stochastic GW background from populations of neutron stars in data from the LIGO & Virgo detectors. By Bose S.* We describe a search in LIGO and Virgo data for a stochastic gravitational-wave background from populations of rotating non-axisymmetric neutron stars in our galaxy and in the Virgo cluster. Employing multi-baseline radiometry, bounds on the GW strain power from these populations can be obtained. These bounds can in turn constrain neutron star equations of state. The current status of the search will be presented. We also assess the expected performance of this search using forthcoming second-generation detectors, including the improvement from locating one of the Advanced LIGO detectors in India.

*Generic black-hole-binary waveform models: issues and progress. By Hannam M., Schmidt P., Ajith P., Bohe A., Husa S., Ohme F., Püerrer M.* Current phenomenological waveform models for the inspiral, merger and ringdown of non-precessing black-hole binaries make use of a single spin parameter, which is an appropriately weighted sum of the two black-hole spins. The errors incurred in making this approximation in GW searches and parameter estimation are in most cases smaller than the errors due to waveform degeneracies between the binary’s mass ratio and the black-hole spins. In generic (precessing) binaries, additional approximate degeneracies, plus the recent insight that inspiral and precession effects can be effectively decoupled, open the possibility of constructing simple generic waveform models. We present progress in constructing such models.

*Approximation methods for bayesian detection statistics in a targeted search for continuous gravitational waves. By Whelan J. , Prix R.* Prix and Krishnan [44] showed that the standard maximum-likelihood statistic used in continuous gravitational-wave searches, known as the F-statistic, could also be interpreted as a Bayes factor using an unphysical prior distribution on the amplitude parameter space. They defined an alternative statistic using physical priors on the amplitude parameters, particularly the geometrical parameters of neutron star inclination and polarization angles, known as the B-statistic, and showed it to be more powerful in the case where the unknown amplitude parameters are drawn from the physical prior distribution. Marginalizing over the amplitude parameters requires a multi-dimensional integral which must, in general, be done numerically. We describe [45] approximation methods which allow analytic evaluation of this integral, allowing the more powerful search to be done without a large increase in computational resources.

*A model-based cross-correlation search for gravitational waves from Scorpius X-1. By Whelan J., Sundaresan S., Peiris P.* The low-mass X-ray binary (LMXB) Scorpius X-1 (Sco X-1) is a promising source of gravitational waves in the advanced detector era. A variety of methods have been used or proposed to perform the directed search for gravitational waves from a binary source in a known sky location with unknown frequency and residual uncertainty in binary orbital parameters. These include a fully coherent search over a short observation time, a search for an unmodeled narrowband stochastic signal, and a search for a pattern of sidebands arising from the Doppler modulation of the signal by the binary orbit. A modification of the cross-correlation method used in the stochastic-background search has been proposed, which takes into account the signal model of a rotating neutron star to allow cross-correlation of data from different times. By varying the maximum allowed time lag between cross-correlated segments, one can tune this semi-coherent search and strike a balance between sensitivity and computing cost. We describe the details and prospects for application of this method to searches for Sco X-1 and other LMXBs. We also present some recent enhancements to the Cross-Correlation search method.

*Construction and validation of multi-mode hybrids obtained from gluing post-newtonian and numerical relativity waveforms. By Calderon Bustillo J.* The construction and accuracy of hybrid post-Newtonian/numerical relativity waveforms of the dominant $l=|m|=2$ spherical harmonic modes has been studied in the past with considerable detail. In this work we generalize to non-dominant modes and study impact and the errors of the procedure from a data analysis point of view.

*Sensitivity of coincident and coherent CBC searches at finite computing cost. By Dal Canton T., Keppel D. * Searches for gravitational radiation from coalescing compact binaries use single-detector matched filters followed by a coincidence stage. An alternative method is the so-called coherent matched filter, where data from all interferometers are combined coherently into a single detection statistic, automatically taking into account the different responses and time delays of the instruments. This method is expected to be more sensitive but also computationally more expensive and has never been used in a blind all-sky CBC search. Important open questions for the advanced detector era are i) at what computing cost the coherent method becomes more sensitive and ii) whether an interesting sensitivity increase can be achieved at a lower cost by combining the two methods into a “hierarchical” search. We address these issues by estimating the sensitivity and computing cost of the coincident, coherent and hierarchical methods under the main assumption of stationary Gaussian noise. We compare the sensitivities at fixed computing cost for different configurations of advanced detectors, including the projected evolution of advanced sensitivity curves.

*Stochastic background of gravitational waves generated by compact binary systems. By Evangelista E.* Soon after the publication of the General Theory of Relativity in its definitive form in 1916, it was noticed that some of its solutions depicted gravitational waves, that is, perturbations in the spacetime which propagate at speed of light and that could in principle be detected. According to such solutions, any mass distributions that underwent some kind of time variation would become a source of gravitational radiation, since at a given moment the spherical symmetry is broken. Thus, from the astrophysical viewpoint, virtually all the processes involving mass deformations and movements, such as stars and black holes, could be considered as potential sources of gravitational waves. Particularly, we are dealing with cosmological compact binary systems in circular and eccentric orbits and the spectra generated by the population of those sources. The main purpose of our work is the formulation of a new method of calculating the spectra generated by such a population during the periodic and quasi-periodic regimes. We used an analogy to a problem of Statistical Mechanics in order to establish the fundamentals of such a method, besides taking into account the time variation of the orbital parameters such as eccentricities and frequencies.
